# Co-design Tensions Between Parents, Children, and Researchers Regarding Mobile Health Technology Design Needs and Decisions: Case Study

**DOI:** 10.2196/41726

**Published:** 2023-04-14

**Authors:** Jason Yip, Kelly Wong, Isabella Oh, Farisha Sultan, Wendy Roldan, Kung Jin Lee, Jimi Huh

**Affiliations:** 1 The Information School University of Washington Seattle, WA United States; 2 Department of Population and Public Health Sciences Keck School of Medicine of USC University of Southern California Los Angeles, CA United States; 3 Ewha Womans University Seoul Republic of Korea

**Keywords:** just-in-time adaptive intervention, JITAI, mobile health, mHealth, participatory design, co-design, children and families, Black, Indigenous, and people of color, BIPOC, child-computer interaction, design, children, mobile intervention, intervention, development, mobile phone

## Abstract

**Background:**

Just-in-time adaptive interventions (JITAIs) in mobile health are an intervention design that provides behavior change support based on an individual’s changing and dynamic contextual state. However, few studies have documented how end users of JITAI technologies are involved in their development, particularly from historically marginalized families and children. Less is known for public health researchers and designers of the tensions that occur as families negotiate their needs.

**Objective:**

We aimed to broaden our understanding of how historically marginalized families are included in co-design from a public health perspective. We sought to address research questions surrounding JITAIs; co-design; and working with historically marginalized families, including Black, Indigenous, and people of color (BIPOC) children and adults, regarding improving sun protection behaviors. We sought to better understand value tensions in parents’ and children’s needs regarding mobile health technologies and how design decisions are made.

**Methods:**

We examined 2 sets of co-design data (local and web-based) pertaining to a larger study on mobile SunSmart JITAI technologies with families in Los Angeles, California, United States, who were predominantly of Latinx and multiracial backgrounds. In these co-design sessions, we conducted stakeholder analysis through perceptions of harms and benefits and an assessment of stakeholder views and values. We open coded the data and compared the developed themes using a value-sensitive design framework by examining value tensions to help organize our qualitative data. Our study is formatted through a narrative case study that captures the essential meanings and qualities that are difficult to present, such as quotes in isolation.

**Results:**

We presented 3 major themes from our co-design data: different experiences with the sun and protection, misconceptions about the sun and sun protection, and technological design and expectations. We also provided value flow (opportunities for design), value dam (challenges to design), or value flow or dam (a hybrid problem) subthemes. For each subtheme, we provided a design decision and a response we ended up making based on what was presented and the kinds of value tensions we observed.

**Conclusions:**

We provide empirical data to show what it is like to work with multiple BIPOC stakeholders in the roles of families and children. We demonstrate the use of the value tension framework to explain the different needs of multiple stakeholders and technology development. Specifically, we demonstrate that the value tension framework helps sort our participants’ co-design responses into clear and easy-to-understand design guidelines. Using the value tension framework, we were able to sort the tensions between children and adults, family socioeconomic and health wellness needs, and researchers and participants while being able to make specific design decisions from this organized view. Finally, we provide design implications and guidance for the development of JITAI mobile interventions for BIPOC families.

## Introduction

### Background

Just-in-time adaptive interventions (JITAIs) in mobile health are an intervention design that provides behavior change support based on an individual’s changing and dynamic contextual state [1]. These digital interventions are unique in that they rely on sensing and mobile technologies to support health behaviors in a person’s rapidly changing environment. Despite the appeal of JITAIs, there remains a gap in the implementation of these interventions [2]. Specifically, few studies have documented how end users of JITAI technologies are involved in their development. Much of the public health research in designing and implementing digital behavior change interventions appears to rely on methods detached from users (eg, ad hoc surveys, researchers, designer-only designs, and randomized controlled trials [3-8]).

In contrast, qualitative co-design methods allow for deeper examination of engagement strategies and persuasion techniques that lead to more effective behavior change and health and wellness. Effective engagement with digital interventions is considered a critical mediator to achieve intended behavior change [9] as greater levels of engagement are likely to be achieved by thoroughly involving user input from an early stage of intervention technology development. Participatory design (PD) is an approach that emphasizes the democratization of design through close collaboration between designers and end users [10]. Co-design is a subset of PD that emphasizes the equitable and equal partnerships between designers and users of technology [11]. In human-computer interaction (HCI)–oriented work, co-design has been integrated in youth web-based safety [12-15], sociotechnical systems for health and wellness [16-18], senior citizens’ needs [19-21], learning technologies [22-25], and many other areas.

Within behavior change intervention research, we have little understanding of how different democratic and collaborative design partnerships could be used for empirical research on JITAIs. Specifically, we aimed to broaden our understanding of how historically marginalized families of diverse ethnic, racial, and socioeconomic backgrounds and acculturation statuses are included in qualitative studies for co-design from a public health perspective.

Only a few studies have documented the design process of sun protection digital interventions [26-28]. As part of our intervention work to improve sun safety among children from diverse backgrounds [29], this study sought to address the following research questions (RQs) on JITAIs; co-design; and working with historically marginalized families, including Black, Indigenous, and people of color (BIPOC) children and adults, with regard to improving sun protection behaviors: (1) What are the value tensions that occur as families co-design new technologies supporting sun-protective behavior changes? (RQ 1), (2) How can co-design methods and techniques be used to center the lived experiences of children and parents in health behavioral intervention projects? (RQ 2), and (3) How do we as researchers respond to families’ value tensions through our design decisions? (RQ 3).

To answer these RQs, we examined 2 sets of data pertaining to a larger study on mobile SunSmart JITAI technologies with families in Los Angeles (LA), California, United States, who were predominantly of Latinx and multiracial backgrounds. Our JITAI app provides real-time technology and delivers tailored educational materials to children on sun safety and diversity in skin color using augmented reality and geofencing.

In these co-design sessions, we conducted a stakeholder analysis through perceptions of harms and benefits and an assessment of stakeholder views and values. We examined qualitative data provided by co-design groups of families in 2 modes: in-person meetings in November 2019 and web-based workshops in May 2020 and June 2020. We co-designed with the families using a specific framework of co-operative inquiry [25,30]. Co-operative inquiry emphasizes that equal and equitable design partnerships can occur between adults (eg, researchers, designers, and parents) and children in the development of new technologies. To analyze our data, we open coded them and compared the developed themes using a value-sensitive design framework by examining value tensions [31,32].

This study makes 3 main contributions. First, we provided empirical data to show what it is like to work with multiple BIPOC stakeholders such as families, HCI or health behavior researchers, designers, caretakers, and children. Second, we used a value tension framework to explain the different needs of multiple stakeholders, how conflicts and tensions occur in the design process, and how we incorporate these tensions into technology development [32]. Finally, we provided design implications and guidance for the development of JITAI mobile interventions for BIPOC families.

### Literature Review

#### Evaluating Digital Interventions With Children

In public health, many studies involving digital interventions are evaluative, focusing on evaluating the efficacy or effectiveness of JITAI innovations for children; only a few have focused on the development of the technology with end users. For instance, in their work on the oral health of children, Jacobson et al [33] developed a mobile app game called Brush Up and conducted a pretest-posttest single-arm study. For children with asthma, researchers developed a JITAI for self-management allowing children to create profile pages; track inhaler use; connect with a care team; access educational materials; and chat with providers, family, and school personnel [34]. Studies on children with autism have also shown the potential of using everyday technologies such as the Apple Watch and web-based peer technologies to help children with autism via scene cues, supportive play, and communication [35,36].

Evaluations of technology designs for mobile health and children also exist as systematic literature reviews. Lau et al [6] conducted a systematic review of 9 information and communication technology–based interventions for promoting physical activity behavior change in children and adolescents, reporting overall positive effects for activity encouragement paired with dietary approaches. Another systematic review involving 34 technology-based interventions for depression and anxiety in children and adolescents found some evidence of benefits using a combination of cognitive behavioral therapy techniques through digital interventions [37].

Despite the number of evaluative JITAI studies, few studies have focused on the user experience and user involvement in the design phase of JITAI technology, especially for those from BIPOC communities. The systematic review by Anderson-Lewis et al [3] of mobile health technology use and implications in historically marginalized communities in the United States revealed that, of 16,270 articles from 2009 to 2016, only 16 studies qualified to implement *mobile health strategies* for health interventions involving marginalized communities. In the review, 9 of the 16 studies focused on populations that identified themselves as Black, Latinx, or Hispanic. Only 7 of the studies focused on Latinx populations.

There are few JITAI studies involving BIPOC communities that focus on children and families together. For instance, to increase influenza vaccine rates in lower–socioeconomic status (SES) children and adolescents, a randomized controlled trial was conducted in which parents received SMS text messages to promote influenza vaccines [7]. In another study, Nollen et al [38] developed mobile health technology for obesity prevention, testing a 12-week mobile technology intervention on lower-SES BIPOC girls (aged 9-14 years) toward increased consumption of fruits and vegetables and lower consumption of sugar-sweetened beverages. In both studies, little information was provided in terms of how and why certain mobile technology features were developed and included. Studies that explicitly describe the JITAI technology design process involving children, adolescents, or their families are extremely scant in mobile health.

#### Developing Designs With Insights From Users

##### Overview

To develop new technologies and designs for health behavior change JITAIs, several methods have been used by mobile health researchers to gather information to support their designs. We argue that there is a spectrum of participation, from no participation to informant design [39] to co-design [25,30]. The following sections provide examples of health behavior change JITAI development processes that demonstrate varying degrees of user feedback (Table 1).

**Table 1:** Dimension showing the range of user participation in methods of design.

Methods of design	Level of user participation, response, and methods
No participation	Literature review, theory, and design guidelines
Usability	Usability testing and A/B testing
Survey methods	Surveys and questionnaires
Informant design	Interviews and focus groups
Design partnership	Engagement in co-design workshops

##### No Direct Feedback From Users

Some behavioral intervention researchers document that they use theory-based approaches and evidence-based guidelines as the basis for their JITAI design. Vidmar et al [8] developed their intervention using addiction-based principles to create W8Loss2Go, a mobile health weight loss app. Other researchers compiled studies through literature reviews to support their mobile health designs. Downing et al [4] consolidated relevant literature related to mobile health and obesity interventions in creating their study on testing the efficacy of personalized short SMS text messages to users through their app, Mini Movers*.* Other studies used literature reviews with expertise interviews without direct feedback from stakeholders. Following a systematic literature review, Jibb et al [5] held an extended conference with 15 experts of varying backgrounds in pertinent fields to provide input on necessary features and feasibility of their smartphone-based pain management app. Once done, the program went through extensive iteration and vetting before being tested out in its first phase with actual users. Finally, mobile health studies that do not work directly with users often will use technology designs from previous research, such as Evans et al [40], who adopted an existing just-in-time messaging app, Text4Baby, to be used for their pilot study to monitor stakeholders.

##### Using Informant Design

PD [10,41] is a design method that involves active stakeholders in the process of innovation and technology development. Even though health behavior researchers claim to engage in PD and co-design in developing their digital interventions, their methods vary largely across studies and often lack sufficient details about the actual participatory process. Bevan Jones et al [42] reviewed 25 original articles and 30 digital technologies to support child and adolescent mental health. Most of the articles and studies reviewed used stakeholder involvement methods such as focus groups, interviews, surveys, expert consultations, questionnaires, observations, and dynamic workshops (eg, design studios, design charrettes, and design jams). The review further demonstrates that some studies involving stakeholder participation use a combination of multiple methods (eg, surveys with focus groups and interviews with workshops).

Informant design uses information-gathering methods such as focus groups and interviews with users and stakeholders as the basis for design [39]. For instance, the development of Jooay [43], a mobile app to facilitate access to leisure and physical activity community programs for youth with disabilities, focused on holding multiple forums concurrently where revisions and testing of the app happened at the same time. The development of a mobile health intervention for the management of type 1 diabetes in adolescents by Cafazzo et al [44] began with interviews with adolescents with type 1 diabetes and their family caregivers.

#### Considerations of Co-design, Race and Ethnicity, and Cultural Characteristics

Co-design research in mobile health is limited to how to best deal with the varying components of the culture and language of end users. Of the 25 studies in the review by Bevan Jones et al [42] of the co-design of mental health innovations, only 2 studies focused specifically on ethnicity, culture, and subgroups. Saulsberry et al [45] documented a culturally adapted, low-cost, primary care internet-based depression prevention intervention. The researchers created 2 adolescent advisory groups, one with adolescents and one with parents (2 Black and 2 Latinx groups), who acted as community advisers. Sobowale et al [46] focused on the design of Project CATCH-IT (Competent Adulthood Transition with Cognitive Behavioral, Humanistic, and Interpersonal Training), a technology intervention to prevent depression in Hong Kong Chinese adolescents. The researchers gathered a sample of 16 bilingual Chinese adolescents and young adults using a series of questionnaires and reviewing 2 modules of Project CATCH-IT.

The benefits of considering ethnicity in PD in mobile health areas have been documented. The comparison by Unertl et al [47] of 5 health informatics research projects using a community-based participatory research approach found that applying these collaborative design methods can be important for those that are typically underserved in health care. Unertl et al [47] found that a community-based participatory research approach to health information and technology design research in underserved communities can be effectively applied. This results in concrete benefits for target communities, especially in the potential for integrating community-based participatory research with user-centered design and PD approaches.

In summary, despite the potential of PD of mobile health technologies with historically marginalized populations, there remains a wide gap in knowledge. First, we have little knowledge of the direct or indirect role of multiple cultures in the design of mobile health technologies. Second, the studies on PD and historically underserved populations do not particularly focus on children and adolescents with their families. We need more research on how bilingualism in multicultural communities involving intergenerational participants affects co-design.

#### Theory

Just as it is important to demonstrate the benefits of PD work for underserved, historically marginalized communities [47,48] in developing JITAI technology to maximize effective engagement with the intervention, it is equally critical to document and analyze some of the challenges in co-designing JITAIs involving BIPOC families. This study relies on the framework of value tensions in design [32] to analyze our participants in co-design. Value tensions in design are grounded in value-sensitive design [31], an interactional approach to understanding the design of values into technology. For example, value-sensitive design entails identifying direct and indirect stakeholders of a sociotechnical system, which takes the approach that emergent values in systems change over time as appropriated by users.

Building on value-sensitive design, value tensions in design look at the method of value dams and value flows. Value dams refer to the design features and policies that are opposed by stakeholders even if they are a small set. Value flows, in contrast, refer to the design features and policies that a larger proportion of stakeholders would like to see implemented in an overall system. Value flows support how to discover the features or setups that bring stakeholders into the system. As value dams and value flows are identified, designers need to consider how to balance the issues that are brought about, even if a small set of stakeholders brings up attention and concern.

We chose to use value dams and flows to examine the dynamics among our participants in the co-design of our JITAI mobile technologies for the following reasons. Parents and guardians, children, and researchers all have their own preferences and ideas about what constitutes concern and harm (dams) and benefits (flows). Designers consider many of the voices in the design by looking at what designs are opposed or supported. Second, value tensions allow us to look at what indirect stakeholders may exist in our design. Although we work with direct stakeholders in co-design settings, it is important to understand who they might consider as indirect and ancillary from within the design and beyond the implementation. Finally, value tensions allow us to look at the organizational policies and practices that exist in the immigrant and BIPOC families in our study. Sociotechnical design is not conceived or implemented in a vacuum. Understanding the stakeholders’ perceptions of the policies of their schools, neighborhoods, and local communities is an important way to examine the values that are present.

## Methods

### Background

This investigation is embedded in a larger study on developing a mobile-based SunSmart curriculum via co-design. SunSmart is our current research attempt to reach children with diverse backgrounds beyond classrooms to increase sun protection knowledge (eg, effects of UV rays on the skin) and behavior (eg, seeking shade and wearing UV-protective clothing) and help overcome barriers to sun safety among these children and their families. Design work on the SunSmart technologies has also been conducted through a set of design-domain experts using *WeDesign* (pseudonym) [36]. WeDesign is an ongoing intergenerational design group at an anonymous location in which children (aged 7-11 years) and adult researchers come together.

### Case Study Design

Through our work with the design-domain children (WeDesign), we triangulated our initial co-designs with 2 groups of BIPOC families in the LA area that represented the stakeholders for our SunSmart technologies. We focused our meaning by acknowledging, confronting, and interrupting racism; facilitating families’ critical consciousness; supporting positive identity development; and being active agents and partners in design.

**Figure 1:**
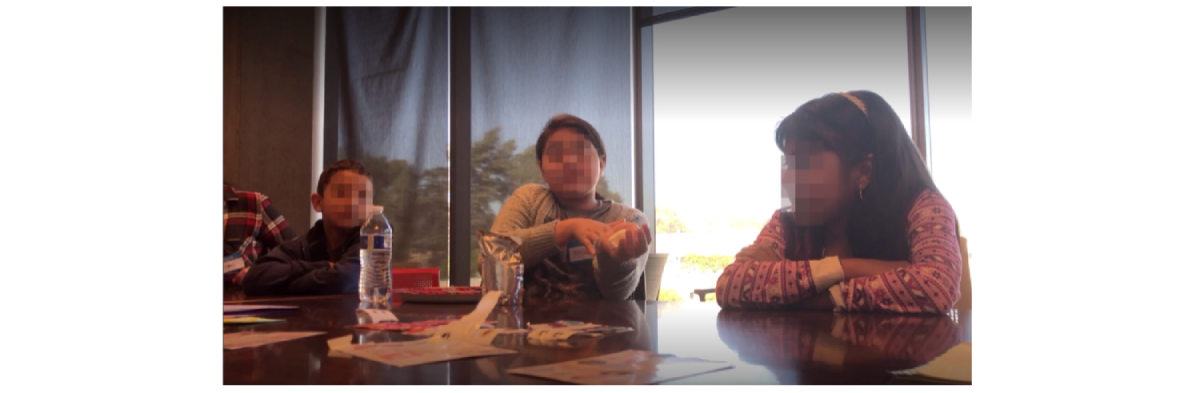
Children co-designing the SunSmart app locally together.

**Figure 2:**
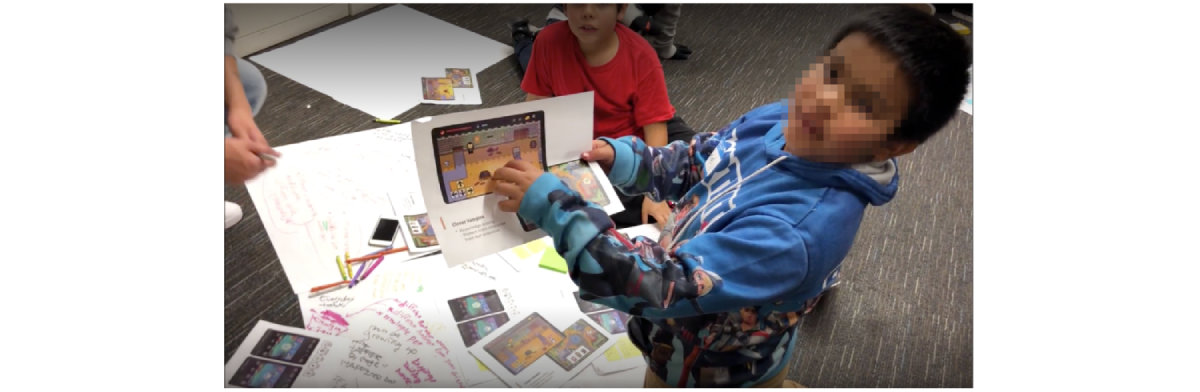
A child working on digital games for SunSmart.

**Figure 3:**
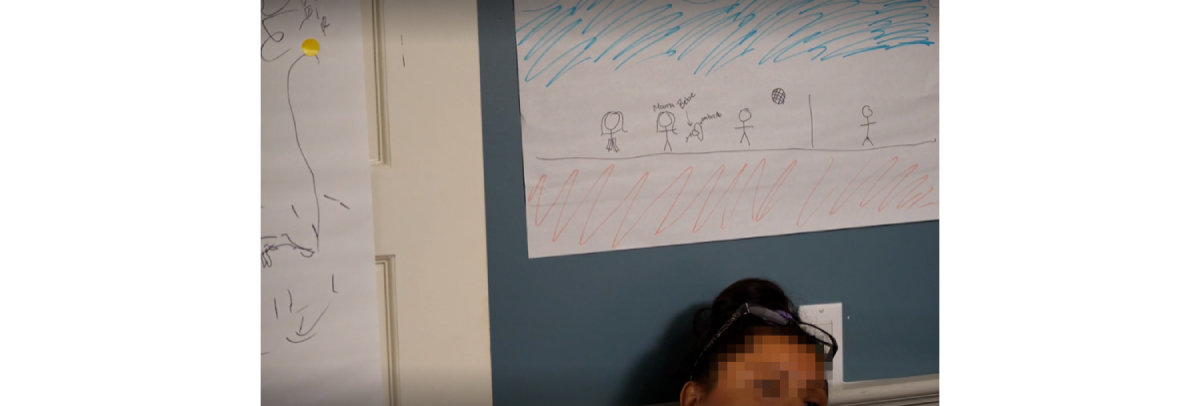
A mother working with other parents on the SunSmart app.

Family design participants are referred to as *subject-domain experts* [49]. We recruited the LA subject-domain expert children and their families from local elementary schools in the LA area (n=29 children; 24/29, 83% Hispanic; n=25 caregivers; 24/25, 96% Hispanic) in November 2019 [49]. In November 2019 (Figures 1-3), we were able to hold 2 design workshops with families. During these first 2 sessions, we collected data such as artifacts and videos of the co-design sessions. We took time to triangulate both the WeDesign (design-domain experts) and LA local families (subject-domain experts) to create the first design iteration of our mobile SunSmart technology.

**Figure 4:**
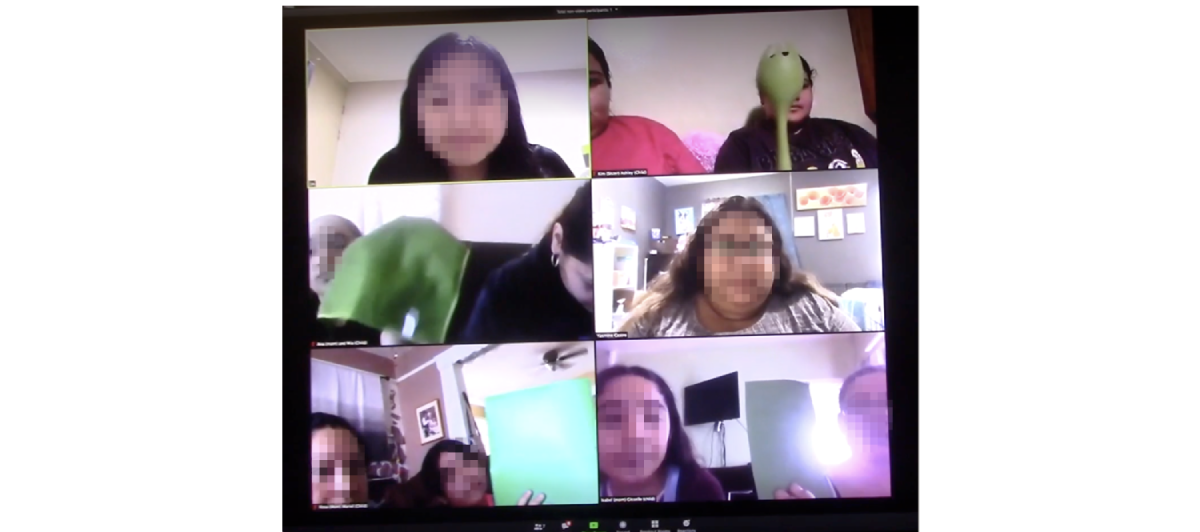
Researchers, children, and parents co-designing together over Zoom video chat (Zoom Video Communications).

After the first design iteration, we invited the groups to return for 2 subsequent web-based sessions to give us feedback on our interpretations of their ideas (Figure 4). However, in March 2020, the COVID-19 pandemic forced all in-person studies to be conducted remotely. We continued to contact the LA families to see if they would be interested in engaging in co-design workshops on the web. Many families from the November 2019 workshops continued their participation in subsequent web-based co-design iterations in May 2020 (n=15 children; 11/15, 73% Hispanic; n=13 caregivers; 12/13, 92% Hispanic) and June 2020 (n=12 children; 8/12, 67% Hispanic; n=10 caregivers; 9/10, 90% Hispanic).

This specific case study focused on the value tensions that exist among the subject-domain expert families. In comparison with our previous studies [49], where we compared the themes between design-domain experts and subject-domain experts, this study is a deeper within-analysis of the subject-domain experts only and the roles and tensions that exist between the different stakeholders (eg, LA children and caregivers) in this group.

This case study is a revelatory case, in what Yin [50] describes as a unique opportunity to observe and examine an unstudied phenomenon. In mobile health for JITAI development, we were unable to identify studies that examined how stakeholders engaged in design efforts and how design researchers made their decisions about the technology. The larger unknown phenomenon examined are the value tensions that exist between different family members as they co-design a mobile health innovation for Latinx families. We selectively chose to study this case for 2 reasons. First, we could work with a group of Latinx American families as a longitudinal case requiring multiple workshops with the same set of families over time (November 2019, May 2020, and June 2020). Looking at multiple families over time (2 in-person workshops and 2 web-based workshops over the course of 1 year) demonstrates how situations and processes change.

### Data Collection

#### LA In-Person Data Collection—November 2019

For the November 2019 local in-person sessions, we collected 2 kinds of data: artifacts from the design sessions in the form of photos and video data of the family co-design sessions. We initially divided the families in the November 2019 sessions into 2 separate groups for the first hour: parents or adults and children. In the parent and adult group, we conducted the co-design session initially as a short focus group to develop trust and comfort with the parents [23]. We chose to separate the children from the parents or adults initially so that both groups could honestly discuss topics that they might not want to talk about with each other. Research in co-design with families also indicates that families, especially parents, need time with their peers and researchers [23] to develop comfort regarding communication of sensitive information.

The initial adult session took place with a Spanish-speaking majority, with 2 researchers (both fluent in Spanish and English) running the co-design sessions. We started with initial questions to the families about their life in the sun and asked them to sketch out how parents encouraged sun protection in their children. Parents discussed their experiences and challenges with sun protection with other parents in a bilingual fashion.

The children session took place in a separate room with design researchers. During this session, the children broke up into co-design groups with 1 adult designer and 3 to 4 children per group. We asked the children to use the “Bags of Stuff” technique [11], in which we provided art supplies, writing utensils, and large paper to create a story about a day in the sun with their families. The children also created low-fidelity prototype technologies to demonstrate how families could protect themselves from the sun. We brought the families back together during the last hour of the workshop. Collaboratively, the children presented the ideas they created during the workshop. The parents proposed their ideas and critiqued the design ideas for mobile and wearable devices, UV protection, and other prototype ideas for their contexts.

#### Web-Based Data Collection During COVID-19—May 2020 and June 2020

We developed the web-based co-design sessions according to Lee et al [51], who noted the need for transparency and improvisation in running sessions through online video chat. On the basis of human participant research protocols, we ran both sessions using Zoom (Zoom Video Communications) web-based meeting software and Microsoft PowerPoint (Microsoft Corp). Using Zoom, we split families into smaller groups (ie, breakout rooms) to have discussions and critiques about the next set of designs. We used Microsoft PowerPoint to share the designs we created and used a series of likes, dislikes, and design ideas for the families to critique the higher-fidelity prototypes [11]. We had families raise up “red objects” for dislikes and “green objects” for likes. After the breakout room discussions, we gathered everyone back for a collective discussion on the entire design process. During these 2 sessions, we collected artifacts through screenshots and by video recording the video chat sessions.

### Data Analysis

Following the subject-domain expert sessions, the investigators had multiple debriefing sessions to synthesize and summarize the main points from each of the workshops. We analyzed the artifacts produced in the workshops (eg, posters, photos, and screenshots) and audio and video recordings. For the audio and video recordings, we reviewed all the data through the development of analytic memos [52] that detailed and time-stamped the collaboration and discussions that occurred both locally and on the web. We decided on key areas to translate (from Spanish to English) and transcribe.

We followed the standards and practices of interrater reliability in HCI [53], particularly with regard to the stance by Hammer and Berland [54] on coding and interpreting qualitative research. Hammer and Berland [54] note the difficulty of traditional interrater reliability using quantitative analysis to justify schemes and coding results. Statistical quantitative analysis of qualitative phenomena can often mask and hide the disagreements and uncertainty about complex data [55]. For instance, developing a coding scheme requires researchers to negotiate and articulate their definitions, mismatches, discussion, and revisions to the scheme [54,56-58]. Along with this process, more insights into the data can arise, leading to productive debates that can help process deeper insights.

Therefore, instead of relying on quantitative counts for agreement, we engaged in the process of consensus coding through an interpretivist lens [54,57,59]. This approach allows for a balance within qualitative work to ensure that researchers do not approach the coding and analysis processes through their own biases and sole interpretations. A primary reviewer of the analytic memos began the coding process using the initial codebook and justifying their coding practices. Using inductive methods to first understand the emerging themes, we initially open coded the analytic memos and annotated the recorded video sessions. For this specific case study, we had 3 researchers look closer at what kinds of challenges and opportunities existed in the design proposals that the families created. Our team coded and gathered quotations and instances to support the themes. For us, the initial coding took place over multiple meetings (N=14 in total from April 2021 to August 2021). The initial codes that we came up with for our codebook included the following: (1) past experiences with the sun, (2) self-awareness about their experiences with the sun, (3) disagreements between children and adults, (4) positive and negative experiences with the sun, (5) features of technologies that parents and children wanted, and (6) ideas they had about the sun and sun protection.

Next, from July 2021 to August 2021, we conducted our consensus process to achieve interrater reliability consistency. Over the following 10 group discussions, we updated the codebook to reflect this initial coding process. Once the primary reviewers completed the first round of coding, the secondary reviewers reviewed the codes and determined the points on which they disagreed. During these meetings, we discussed disagreements and refined the codes. The primary goal of these meetings was to come to an agreement on coding and yield initial concepts and themes (eg, repeating concepts, topics, and meanings). The coding process involved meeting, diverging, synthesizing, highlighting, revisiting memos, and eventually negotiating. If a secondary reviewer disagreed with the primary coder, we worked together as a team of 6 researchers to come to a consensus on whether the coding had been applied properly.

Once primary and secondary coders came to a consensus, along with a finalized codebook, we proceeded with axial and selected coding [60] and produced larger themes. From these initial themes, we generated three major themes: (1) experience with the sun, (2) misconceptions about the sun, and (3) technological needs. Deductively, we went back to value tensions and value-sensitive design to make sense of the large themes [32]. Qualitative researchers can start with an inductive coding process and finalize their approaches using a deductive process [61]. Using a hybrid inductive or deductive approach, we compared our initial inductive themes with what we believed were deductive value dams (challenges to the design) and flows (opportunities for design) in the quotations and memos. Although not mutually exclusive, we also coded instances of mixing value dams and flows together. Finally, as a reflection process in this case study, we mapped our design decisions regarding the app to the value dams, flows, and dams or flows.

### Ethics Approval

We performed all procedures in studies involving human participants in accordance with the ethical standards of the institutional or national research committee and the 1964 Helsinki Declaration and its later amendments or comparable ethical standards. All research procedures were approved by the University of Southern California Institutional Review Board (HS-18-00329) and the University of Washington Institutional Review Board (STUDY00003744).

### Informed Consent

We obtained informed consent from all adult individual participants included in the study, and an assent form was obtained from all children participants included in the study. We provided translations into Spanish for adults and explanations of privacy, confidentiality, safety, and risks. During the consent process, we indicated that both parents and children were free to withdraw at any time.

### Privacy and Confidentiality

All qualitative data were anonymized for the analysis and stored on a secure password-protected server (Health Insurance Portability and Accountability Act and Family Educational Rights and Privacy Act compliant). We have blurred the faces in the photographs. All names of the children and adults provided in this manuscript are pseudonyms.

### Compensation

We financially compensated all families for their participation in this study. Each family received a gift card of US $30 for each co-design session in which they participated, whether web-based or local.

## Results

### Overview

This case study is written as a narrative with major themes. Narrative case studies are used to understand the stages and phases in the process and investigate the phenomenon of interest within its context [62]. The narrative format captures the essential meanings and qualities that are difficult to present, such as quotations in isolation. The narrative case study provides information with context to make it more accessible for the reader. Vignettes and narrative forms have been used in medical research and public health work to convey sensitive information in context [63-65].

**Figure 5:**
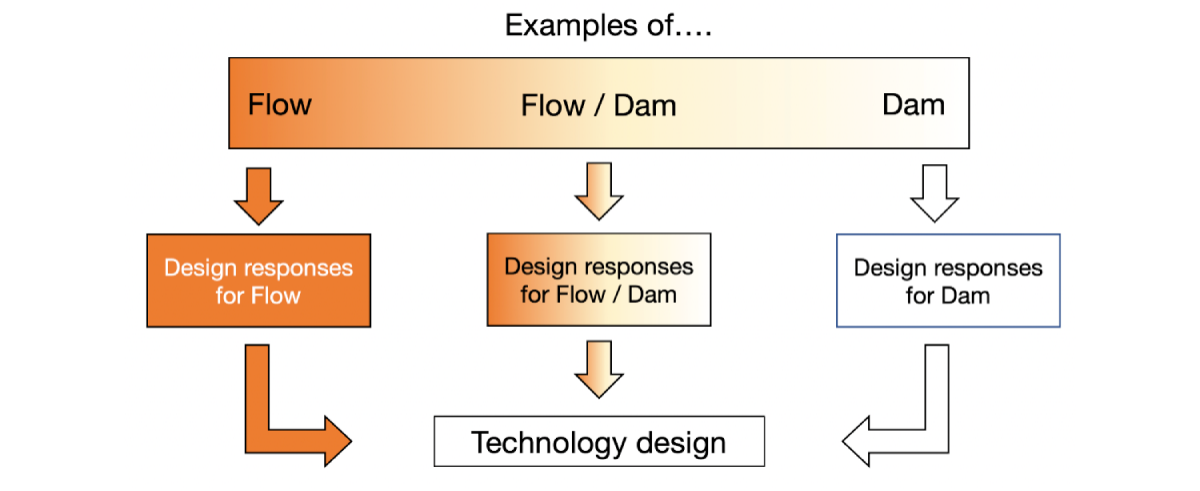
Diagram of the framing of the findings in our co-design with families.

For this case study, we present three major themes from our co-design data: (1) different experiences with the sun and protection, (2) misconceptions about the sun and sun protection, and (3) technological design and expectations. For each of these themes, we also provide value flow (opportunities for design), value dam (challenges to design), or value flow or dam (a hybrid problem) subthemes. Finally, for each subtheme, we provide a design decision and a response we ended up making based on what was presented. See Figure 5 for the framing of our findings. For simplicity, we have labeled the adults as Name (adult) and the children as Name (child). We provide direct quotations from our participants as examples from our coding scheme. When possible, we provide both the English translation and original Spanish.

### Theme 1: Different Experiences With the Sun and Protection

#### Value Dam: Wearing Sunscreen Every Day Is a Hassle, and Families Tend to Dislike Sunscreen

##### Overview

When planning for outings with the sun and skin in mind, the families in our study differentiated between everyday preparation and special day preparation. An example of everyday preparation came from our in-person co-design session. Rosa (adult; parent) shared her personal experience with our group on how she takes care to put sunscreen on. Rosa knows to carry sunscreen around as she knows how sensitive her skin is to the sun. At the same time, her husband Mario (adult) indicated that he did not like the stickiness of sunscreen or how the opaque white cream that would cover his face seemed like it was part of a Halloween costume. Many members of the families expressed the same opinion that sunscreen is not pleasant to wear. Long-sleeved clothing is also hot and uncomfortable and particularly bothersome to carry around.

Rosa’s family once went to Santa Monica Beach, where it was cooler than the city. Although Rosa wore sunscreen, Mario did not put sunscreen on because of how sticky it was and because he felt embarrassed that the sunscreen application looked like poorly done makeup. While his family went into the ocean, Mario laid on the beach for a while. This decision proved to be problematic. At night, Mario could feel the sunburn, especially after hot water hit his skin:

I felt horrible. It started to peel after a week...And now I cover myself well. (Me sentí horrible. Ya como a la semana me empezó a pelar...Ahora ya me tapo bien.)

Mario; adult

He would never have really thought about it as his skin tone is darker and he does not typically become sunburned. Mario mentioned that his brother’s skin is lighter in tone and sunburns easily. He recalled touching his brother’s sunburn and his brother yelling in pain and irritation. After the Santa Monica Beach experience, Mario understood better. He noted that, because of that experience, even when it is cloudy, he protects himself more.

##### Our Design Response

**Figure 6:**
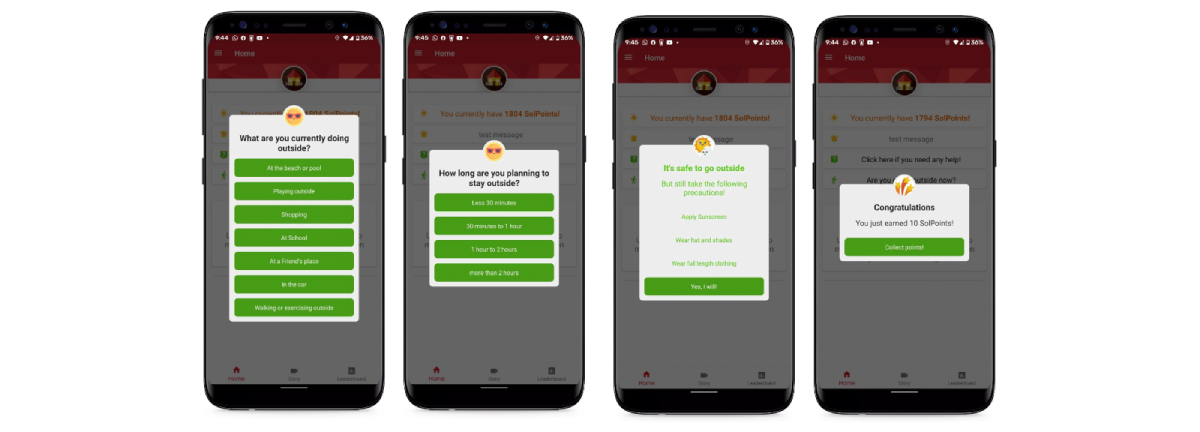
Our SunSmart app provides brief reminders to protect users from the sun based on a geofencing algorithm.

For our mobile technology, as families disliked sunscreen, we provided alternatives for them to consider regarding sun protection. Using a geofencing algorithm, we recommended these alternative methods only when participants were detected to be outside for a certain duration that exceeded a personalized threshold, calculated based on the child’s individual skin phototype. We also provided reminders of various sun-protective methods: protective clothing, seeking shade, and wearing a hat and sometimes sunscreen at the time of acute UV exposure (ie, being outdoors for longer than the prespecified duration based on individual sun sensitivity; Figure 6).

#### Value Dam: Preparation in Advance for Adequate Sun Protection for a Given Context Appears to Be a Moving Target

##### Overview

Tensions came in the form of families being either overly prepared most days (which was burdensome) or underprepared for special days (which could cause physical burns and heat-related problems). Even strategies that worked in one context did not always work well in another. A family member noted that everyday preparation did not always work as well on special occasions (eg, vacations). Felicia (adult) mentioned that, when they usually go to the beach in LA, sunscreen with sun protection factor (SPF) 40 is fine, but the same sunscreen in Northern Mexico does not prevent sunburns. She had extremely bad sunburns on the first day after applying SPF 40 and eventually had to switch to SPF 60 to prevent further burns on her skin in Mexico:

I use the same [SPF] over there [in Los Angeles] and it works, but here [Mexico], it’s different. The sun is different. I didn’t know [that].

Felicia; adult

Felicia noted that the area in Mexico that they were in had more sun exposure than that in LA and that sunburns occur differently in different places.

##### Our Design Response

**Figure 7:**
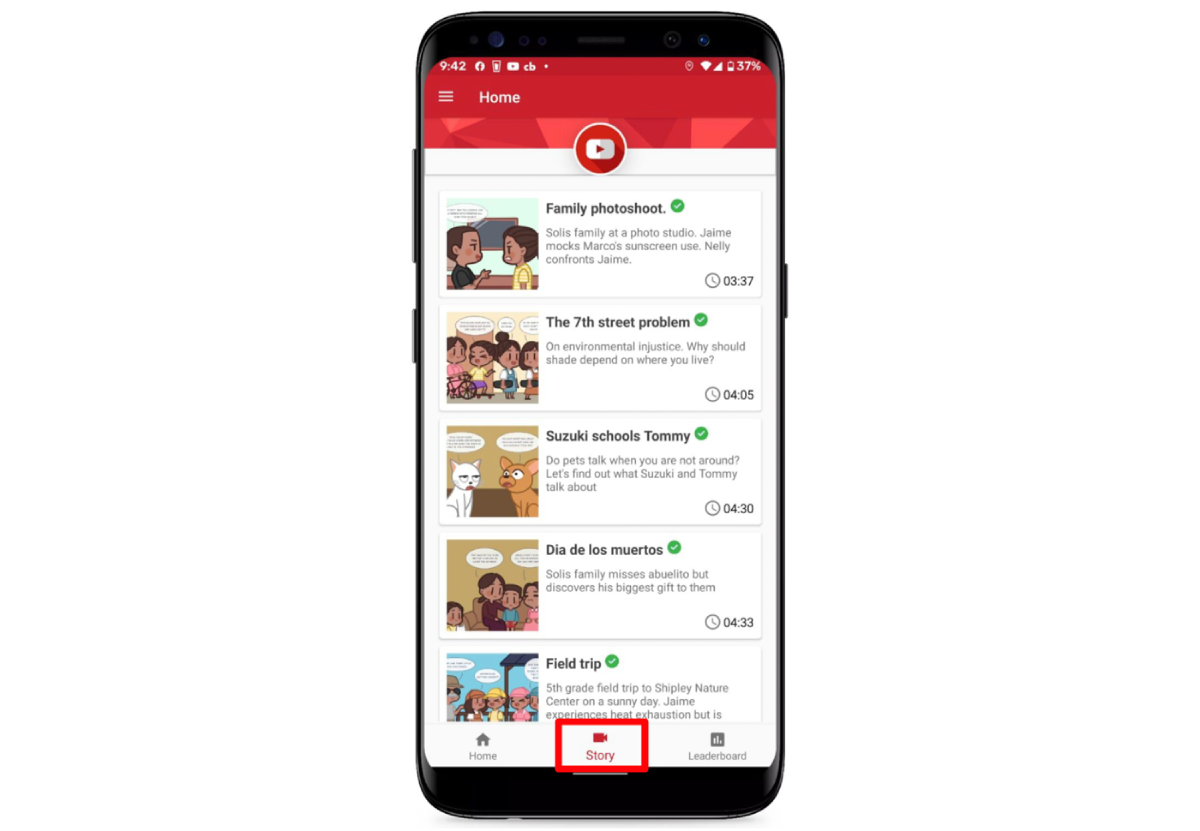
The SunSmart app features stories about Marco and Nelly with complex scenarios about the sun to consider.

As optimal sun protection depends on context, we folded these complex scenarios into our “Marco and Nelly” story (Figure 7). “Marco and Nelly” is a 7-chapter story about local siblings who live in LA with their family and friends in which our sun safety education materials were embedded in a narrative format. We provided these story-based scenarios for families to consider, such as their ideas regarding skin tone, sun sensitivity, various methods of sun protection, role modeling, and contexts. We also chose to help the children understand their context by asking them brief questions about what they were doing at the current moment upon receiving JITAI prompts. By knowing more about their context, we were able to provide better and more focused advice to support sun safety.

#### Value Flow: Some Families Do Prepare for Intense Days, Specifically Special Outings

##### Overview

In contrast, families prepared for special days such as outings, vacations, and especially sun-intense days. For our session with LA local families, Maria (adult; mother) recalled a specific incident when her family was unprepared for their specific outing. She said that her daughters and husband went for an unplanned walk in San Fernando. The temperature was approximately 41 °C, and as they had not anticipated the weather or the walk, they were wearing the wrong shoes, which caused their feet to burn:

And then my husband told my daughter, “you’re crazy you brought me to burn my feet.” (Y mi esposo le dijo a mi hija “tu estas loca me trajiste a quemar las patas.”)

Maria; adult

The whole family faced difficulty because of the sun and the hot weather that day. They later bought sunscreen and long-sleeved shirts for protective measures and some ice cream and desserts to cool off. Even under such extreme circumstances, the sunscreen was too sticky for the liking of the daughters (value dam).

##### Our Design Response

We designed the system as if there was some knowledge of sun protection. Some of our “Marco and Nelly” stories included these special days and outings for the families to consider. As families had existing knowledge of sun protection, we chose to focus more on embedded survey questions to help us understand what they already knew about sun protection.

### Theme 2: Tensions Regarding Misconceptions About Sun Protection

#### Value Flow or Dam: Families Have Extensive Knowledge of Sun Protection, but They Also Have Misconceptions About How Sun Protection Works

##### Overview

Families in this study did not have a deficit in knowledge of sun protection strategies. Instead, they had a wealth of knowledge of sun protection strategies that we could involve them in. However, families did have some alternative ideas about why sunburns occurred. For example, one of the parents thought that sunburns could be amplified in the ocean or commented that their sunscreen did not work after going into the water. For instance, Tania (adult) noted that she had a bad experience with sunburns when she went to Guatemala and swam in the Atlantic Ocean. She became sunburned in the ocean water even though she had put sunscreen on before leaving her home. Tania explained that, when it comes to sun protection, she went above and beyond, taking hats, umbrellas, and anything that would protect her from the sun as she develops sunspots when she spends extended amounts of time in the sun.

During Tania’s trip to Guatemala, she put on sunscreen before swimming. However, she still became sunburned and thought that the saltiness of the ocean caused the sunscreen not to work. Another parent acknowledged that sometimes, when they put waterproof sunscreen on, it is not actually waterproof or protective. Tania agreed, saying that sometimes she waited some time before going into the water after putting sunscreen on, but that that has failed to work for her:

I stayed with my [sunspots]. They peeled on their own a lot, [and I] couldn’t sleep, [so I] put on lotion. From the house until we get to the water, nothing happens. So there should be a waterproof [sun]block, right? (Me pele muchísimo, no podia dormir entonces les eche crema...Yo piensó que de la casa hasta que uno llega a la agua ahí no pasa nada. Entonces tendría que haber un bloqueador que es impermeable no?)

Tania; adult

The tension lies in noting how to acknowledge the funds of knowledge and depth of understanding families already have of sun protection while at the same time not punitively correcting families on any misinformation and misconceptions they have about sun protection behaviors.

##### Our Design Response

**Figure 8:**
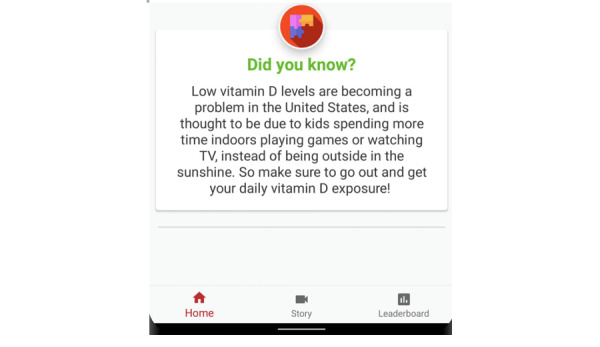
The SunSmart app uses Did you know? facts to help families learn about sun protection.

We did not want to be condescending in our design decisions to the families. Tania’s observations of developing sunburns in ocean water are consistent with what we know of sunburns. However, the salinity of the ocean is not the reason why sunburns happen. Likely, the ocean water may have washed off the sunscreen, or the sunlight reflected off the water with more intensity. Instead of correcting families’ knowledge and presuming deficiency in knowledge, we added a “Did you know?” series of facts that show up in the design (Figure 8). The facts do not tell the parents and children what is right or wrong. Instead, we chose facts that allowed for conversation starters with the app.

#### Value Flow or Dam: Community Experience Not Only Can Support Families in Sun Protection but Can Also Create Some Conflicting Information

##### Overview

Parents in the second round of web-based co-design sessions explained that many people in the Latinx community think that they do not need sun protection as some are darker-skinned than other groups of people. However, a parent mentioned that she believed that more people in her Latinx community need awareness of skin cancer protection. Carmen (adult) believed that many people in her community do not think of themselves as susceptible to skin cancer:

I use makeup so sometimes I only put on a beauty cream that says it has solar protection supposably a kind but I don’t use sunscreen not even Neutrogena. (Yo uso maquillaje entonces a veces solo me hecho una crema de belleza que dice que tiene protector solar según pero no uso protector solar, ni Neutrogena.)

Carmen; adult

The Latinx community tends to have darker skin pigments, so some of the families may not pay as much attention to their skin’s sensitivity to the sun as members of other communities might. Indeed, although the risk is lower, there is still a risk of skin cancer in the Latinx community, and poorer outcomes have been extensively documented for ethnic minority groups, including this community [66].

Similarly, community experiences played a part in how families thought that, as they were of Latinx descent, some of whom tend to have darker complexion and skin tones, they were generally not affected by skin cancer. At the same time, there was acknowledgment from the families that darker skin was not the end-all for sunburns. The experience associated with sunburn and exposure ranged from none to annoying to painful, and we noted that it was difficult to assume a single experience and understanding of sunburn and exposure. This finding is especially critical to consider within the context of designing an intervention targeting children from diverse backgrounds. Families indicated to us that the degree to which they had poor experiences with the sun, pain, and skin sensitivity was different for each person, even within a family. For instance, some children with darker skin tones had little direct experience with sunburns. Jack (child) said that he had never experienced a sunburn but knew someone who had. In total, 7% (2/29) of the children noted that sunburns were mildly irritating. Ryuo (child) said that he developed a sunburn on his nose and that it was annoying rather than being very painful. Gissellae (child) talked about very painful sunburns, saying that “it hurt because it kept bugging me.”

##### Our Design Response

**Figure 9:**
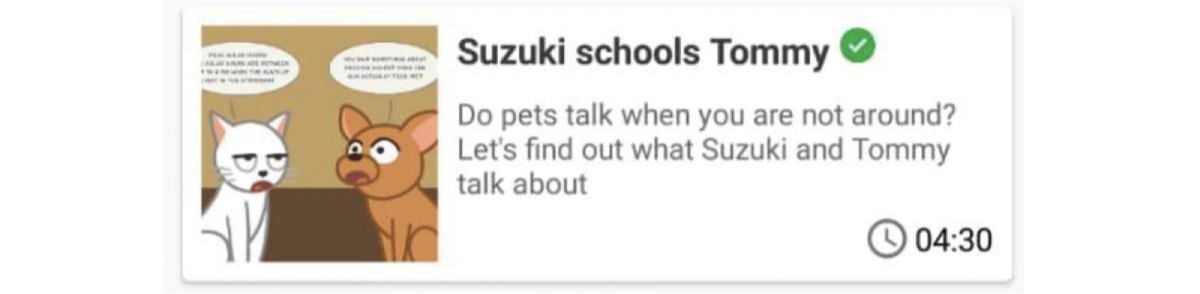
The SunSmart app uses animals as story characters to talk about skin tones and sun sensitivity.

A range of experiences were told as part of the “Marco and Nelly” narrative, in which the story’s characters with various skin tones and sensitivity to the sun were introduced as they went about their daily lives. As culture and ethnic background can be sensitive topics, we also used animal characters to help children understand skin tone and varying degrees of skin tone sensitivity to the sun (Figure 9).

### Theme 3: Technology and Design Concerns

#### Value Flow or Dam: Questions About Including Gender Into the Technology

##### Overview

Families in this study had several concerns regarding the design of the technology. Parents and children each had their own separate concerns, with some overlapping issues. Some parents in our study (3/25, 12%) had specific concerns about the SunSmart technologies. Parents noted that the gender of their children may need to be addressed. For instance, one of the design features we had considered was a daily poll question that would be pushed out to the devices each day. Johanna (adult) in the remote co-design session mentioned that she wanted the questions from our technologies to be based on gender. She wanted us to be aware that the same daily poll questions could not be asked to every child. For example, Carmen (adult) mentioned that asking a girl about popular boy shows and characters would decrease engagement for the child on the app. She wanted the technology to be catered to each child’s gender as she wanted the children to feel more comfortable on the app and engage with it as much as possible:

We need to consider gender, so if we’re asking boys about popular boy shows or boy characters then we should ask girls about popular girl shows or characters that are popular among girls.

Carmen; adult

As we intended to be inclusive of all children, we had the “other” category available for the child’s gender when we were verbally collecting intake information for our co-design sessions. A parent expressed her concern—“Whoa, don’t give my child any ideas (about gender)”—which caused a few other parents nearby to laugh along. During the co-design sessions, we realized that this was a highly culturally sensitive issue.

##### Our Design Response

Considering the sensitivity regarding the topic of gender, we opted to have the survey item options to be as follows: “I am a boy” (with a generic emoji); “I am a girl” (with a generic emoji); and an option button that said, “I’ll tell you later.” We chose not to have gender-specific design features because of too much variability, and correlation with gender was not necessarily causation for SunSmart behavior.

#### Value Flow or Dam: Children Wanted More Fun Features (Gaming and Personalization), Which Seemed to Not Be a Priority for Their Parents, With Some Worried That the Fun Would Be Too Distracting for the Children

##### Overview

Families in the in-person co-design session noted that they were worried that the SunSmart designs would be too gamified and that the lessons that we were trying to present to the children would be lost. Although children did focus a lot on the engagement and fun factors of the technology, both parents and children did want to make sure that the technological design was accessible. The families noted that *solar paper augmented reality* design was not very amusing as it was not straightforward in terms of instructions. A child talked about the initial designs as being boring and confusing, whereas the parents did not know whether the designs were too focused on science, technology, engineering, and mathematics learning and not enough on sun safety. Children also wanted to add learning games to make the technology more fun. A child recommended that the solar paper activity be used “for a board game because it’s light*.*”

Although parents focused more on concerns about the technology (eg, gender, bullying, and learning), children in our co-design sessions focused more on the fun, interaction, and customization of the technology. In the in-person co-design session, the children also had knowledge and strategies for promoting sun protection among themselves. For instance, the children explained to Jason (adult; researcher) a clever vampire game concept. They noted that vampires could be good characters to express sun safety behaviors, learn the relationship between sun exposure and protecting the skin, and eventually be role models. In this game, the vampires would have to have multiple methods of being sun-safe, such as hiding from the sun and wearing protective clothing. One of the children suggested that the vampire could adapt to different environments such as “resources in the [virtual] village.”

**Figure 10:**
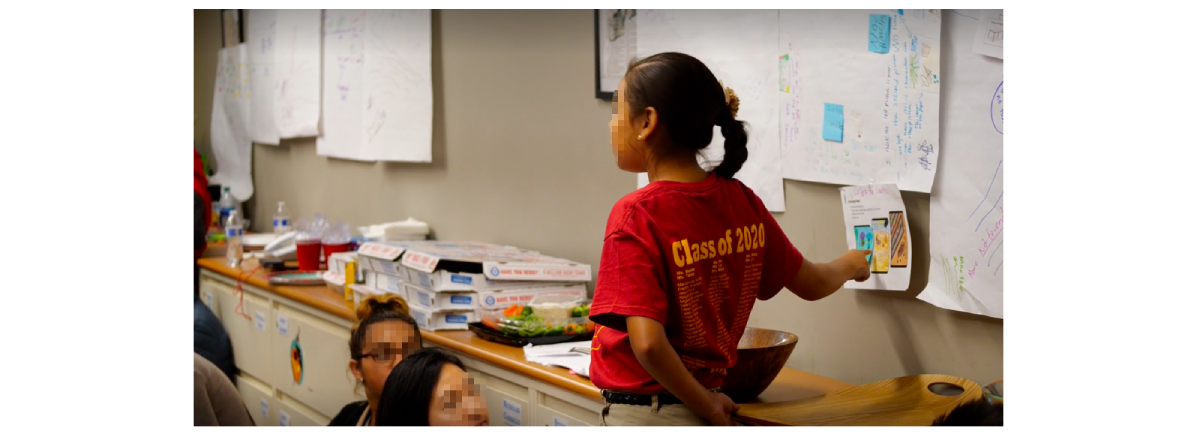
Child presents their designs to other children, parents, and researchers.

Children also thought about the designs as possibly resembling the digital games they had experienced (Figure 10). Some of the boys in the LA local families wanted games about SunSmart technologies to resemble Fortnite with moving images and videos. Some of the children in the LA local families presented how they wanted to see the character live and obtain more features instead of just living and dying. They also wanted to see mini games. A child in the in-person session elaborated on how they did not want to see the digital Tamagotchi-like character die but rather wanted to see it grow up and face challenges. The children in this scenario wanted the game to have more animated fun components. They wanted the character to have more features and face challenges, and they wanted to be able to play mini games instead of the simple game that existed currently.

Children also asked for customization and personalization. In the in-person session, one of the children thought of making images into an emoji and having an option to edit things. They wanted the ability to add specific captions and editing. Tristen (child) said that the children thought that the images could have been drawn better, and they did not like the time element. Daniel (child) wanted to improve the storylines in each picture.

However, parents had concerns that the game component of the technology would overtake any particular interest in or learning about sun safety. In one of the LA families, parents mentioned that the idea of a Tamagotchi digital character to be taken care of by the children would be distracting from SunSmart lessons. They were concerned that the children would focus too much on the digital caretaking game rather than on learning the intended sun safety lessons:

They (children) do a lot for their pet but that [behavior] would not necessarily translate to themselves.

Nobu; adult

Parents in this study also wanted more specifics on whether the technology could be used to help with sun protection. One of the parents in the remote co-design session said that they voted green as she thought that it was a good idea to know how much protection each sunscreen provided and that it would be nice if the app could also give specifics on how much sun protection a person needs based on where they are and where they are going. Parents wanted the app to cater to an individual person’s skin color and tell them what type of sunscreen or protection they would need. This form of personal design would help each individual person who uses the app.

##### Our Design Response

**Figure 11:**
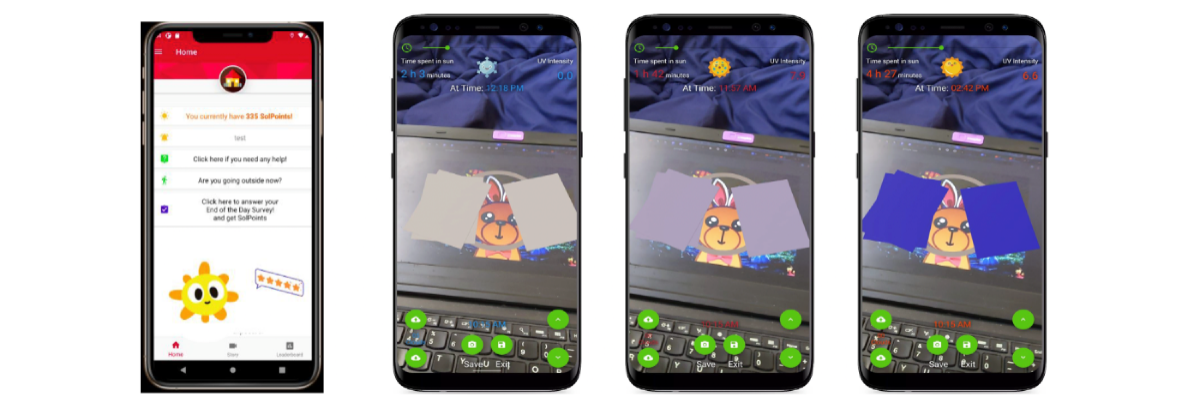
The SunSmart app features a digital sun character and more dynamic augmented reality activities to teach about UV light.

To respond to parents’ and children’s concerns about how uninteresting the augmented reality design could be, we integrated Tommy, the dog character from the “Marco and Nelly” story, into the solar paper augmented reality exercise. This slight change made the exercise less dry and more dynamic than just testing paper with the sun. We created a sun character to encourage children to increase their daily interaction with our app and complete the educational tasks. Considering the parents’ concerns about the app being too gamified, we chose not to make it so that the children did not need to keep the sun character “alive” (Figure 11).

#### Value Dam: Families Were Worried About Cultural Appropriateness in Technology Design

##### Overview

In the in-person session, some children remarked that the technology regarding sun protection could have elements of Lotería, a traditional Mexican game of chance based around a 4 × 4 grid. It has similarities to other children’s games such as Bingo. However, some of the girls in the study stated that the SunSmart technology should not resemble Lotería. They did not want the modern SunSmart technologies to integrate it as it was related to their childhood and traditional customs. The girls who voted for the Lotería game wanted to keep it the same, but if it were to change, then it should be called Bingo:

This is our childhood. This is important to us. If you want to make changes, call it Bingo.

Lisa; child

Other feedback from children suggested that the technology design use word guessing games.

##### Our Design Response

**Figure 12:**
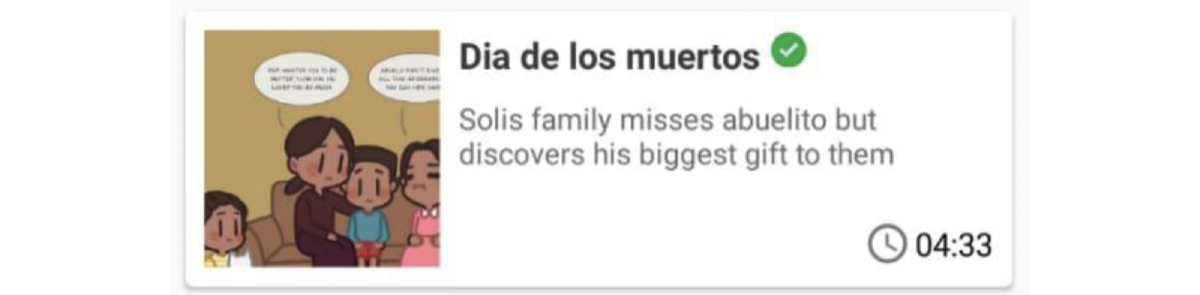
The SunSmart app included traditional holidays into the story line as a way to highlight visibility of cultures.

There is a very fine line between cultural appreciation and appropriation [67]. Although we were engaged closely with Latinx families in the co-design, we ultimately chose not to highlight the Lotería game in the app. Our decision was based on the idea that, although some families could appreciate the integration of Lotería, it could have also offended some families. Lotería is a game that is traditionally played in a specific holiday and family context. We did not think that SunSmart technologies were part of that traditional context. Ultimately, we chose not to integrate it into the design. Instead, cultural appreciation in the design came in the form of storytelling. We chose to integrate cultural holidays and events into the main storylines to demonstrate visibility (Figure 12).

#### Value Flow: Both Parents and Children Had Healthy Concerns About Privacy and Security

##### Overview

Both parents and children were generally concerned about the digital app passively collecting data and analytics about the children. Specifically, Nobu (adult) in the in-person session brought up the idea of trackers, cookies, and cross-referencing data that would target children with digital advertisements. We also came up with the design of an app that would allow families to take photos of a sunscreen bottle and see information specific to that sunscreen. Nobu thought this was not a good idea because of privacy concerns:

You always worry about kids on any app, who’s under 13. That’s why I prefer parent-child apps...because [kids] give up so much privacy at the click of a button.

Nobu; adult

The children thought about privacy as well. Bryandon (child) said that he liked the idea of creating individual user accounts as he could mask his identity by creating his own username without his name in it. However, another child mentioned that they did not like creating usernames as they did not want their name tracked down. This specific child was concerned about how creating their username in this app would affect their privacy as, many times, usernames can be a way for hackers to track down who the person using the app is.

##### Our Design Response

**Figure 13:**
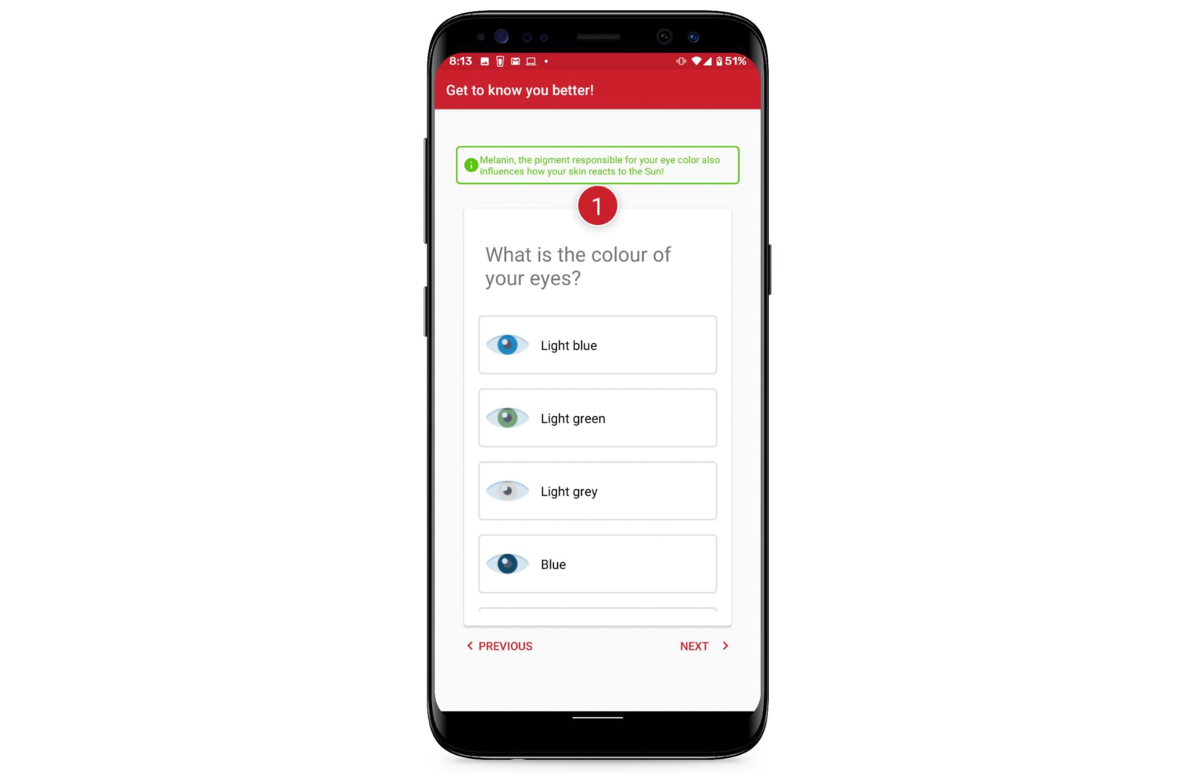
We included an Information button to further explain why we collected certain personal data (eg, eye color).

We wanted to make sure that privacy and security were important design features in our app, especially as it concerned health. We abandoned the sunscreen bottle detection feature that would indicate the features of the sunscreen but, ultimately, store data on their use. We also encouraged the children to use nicknames on the leaderboard to protect their privacy and confidentiality. We included an “Information” button to further explain why we collected certain personal data (eg, report of eye color as certain eye colors are more sensitive to light). For example, children thought that it was strange to ask for the eye color as it is private information. We added explanations as to why these data were collected to be more transparent about why we collected certain data (Figure 13).

## Discussion

### Principal Findings

#### Overview

In this case study, we examined the value tensions of mobile health co-design in families in LA over the course of November 2019 (in person) and May 2020 to June 2020 (on the web). On the basis of the interactions and designs of the families and children, we were able to map out the value tensions they brought up (flows, dams, or both) and connect our design decisions on SunSmart mobile health with these design values. Figure 5 summarizes our approach to co-design, value tensions, and design decisions. In the following sections, we present our discussion by answering our 3 RQs and tying this work to future studies.

#### RQ 1: What Are the Value Tensions That Occur as Families Co-design New Technologies Supporting Sun-Protective Behavior Changes?

Although many HCI and design researchers have demonstrated the complexity of stakeholders in how they make decisions for their digital designs [22,25,68], there is far less work that highlights such complexity in digital health behavior research to make design decisions about JITAI and mobile health innovations. Design strategies that do not involve direct human feedback and input include using guidelines [8], literature reviews [4,6,37], and previous designs [40]. Behavioral interventions that do include direct feedback have often used interviews and group interviews to solicit advice on design [5]. Finally, some health behavior researchers do use PD methods such as design workshops [69,70], advisory groups [45], and co-design teams [26,27]. However, most of this work in mobile health does not address issues of designing with historically marginalized families with children and adolescents, especially when it comes to considerations of cultural identity.

In this study, we observed value tensions that existed in how families conceptualized their own health practices and experiences with technology and cultural backgrounds with respect to sun safety practices [32]. We demonstrated that previous design theories in user-centered design and HCI can complement work on innovation and intervention design in mobile health. Specifically, we believe that using value tensions in collaboration with historically marginalized families and children is of major importance for digital health behavior interventions [32]. Through deeper dives into the co-design process, we advocate for more transparency in mobile health, JITAIs, and health behavior intervention researchers and designers. This is especially true when working with diverse populations to improve public health, where the values of historically marginalized communities must be reflected in technology design. The contribution of our work is that we demonstrated the complexity of designs when working with diverse populations in public health. It is especially difficult to balance the multiple stakeholders’ voices with value tensions. Overall, values matter to people in sociotechnical systems such as JITAI mobile technologies. We as design researchers and public health experts need to understand that, at times, there are unresolved value tensions that have contributed to the failure of numerous technology implementations to support behavior change.

#### RQ 2: How Can Co-design Methods and Techniques Be Used to Center the Lived Experiences of Children and Parents in Health Behavioral Intervention Projects?

The use of co-design and value tensions helped us better understand the skepticism of families toward outside public health researchers [25,30,32]. The literature on the mobile health and public health space demonstrates that historically marginalized families mistrust academic institutions. For instance, Liu et al [71] note that, for lower-SES populations, there are specific mobile health barriers such as fluency with mobile apps, limited health literacy, lack of empowerment, and historical mistrust of health care systems. As mobile health platforms are becoming more pervasive in health care delivery, the amplification of digital divides will make accessibility disparity worse. Reflections on community-based participatory research on the development and deployment of mobile health need to focus on equitable partnerships in designing and seeking solutions; public health concerns; and how race, ethnicity, racism, and class influence health outcomes [72]. Similarly, it is important to acknowledge that mobile health technologies cannot be developed in isolation without a deeper understanding of the structural conditions of people’s lives [72].

Through co-design [30], we believe that there is a way to support families’ engagement in research and design. The families in our study, although noting healthy skepticism of mobile health technology (eg, privacy, security, cyberbullying, screen time addiction, and learning and growth), also wanted to still be a part of our workshops over the course of a year. Several families noted that this was the first time they had engaged with researchers in this capacity and wanted to continue to meet with us through video chat even during the peak of COVID-19. We noted the tension of challenging the design of technology for their communities, but these families also felt strongly about and took pride in being part of the design process.

Finally, we want to consider the value tensions in family knowledge and technology use. The co-design workshops in mobile health also show how previous and new knowledge of sun protection makes design complicated. Intrafamily knowledge focuses on what families think about their experiences within the family [73], such as differences in skin tone or sensitivity to the sun across members of a single family and how each individual family thinks about sun protection during vacations, extreme events, and everyday life. Interfamily knowledge is about how families consider their sun protection experiences compared with other families in their network [74]. For instance, some tensions we observed focused on how families thought about how sun protection affected other families (eg, Latinx American or multicultural families with darker vs lighter skin tones). Finally, intergenerational knowledge highlights role modeling and how children’s knowledge and design insights compare with those of adults [75]. Children in this study had different design priorities (eg, fun, gaming, and character development) from those of the adults (eg, privacy, security, and cyberbullying).

Overall, the reasons technology adoption and behavior change through mobile health and JITAIs are much more complicated, but we argue that it is necessary to understand the push and pull tension that the multiple competing stakeholders (eg, parents and children) may have. We note that traditional methods in public health (eg, surveys, focus groups, literature reviews, theory adherence, and experimental design) need to be complemented with user-centered design [11], specifically in how co-design and PD can provide a deeper understanding of families [10,23,25,30].

#### RQ 3: How Do We as Researchers Respond to Families’ Value Tensions Through Our Design Decisions?

Technology adoption and behavior change are difficult processes. The systematic review by Dugas et al [76] of 21 articles (published between 2007 and 2017) reporting randomized controlled trials of mobile health interventions concluded that, although mobile health technologies (eg, apps and wearable interventions) are becoming more personalized, the effects of mobile health interventions on behavior change are still inconsistent. As such, there are opportunities for improvement. One such opportunity focuses on better understanding individuals with different person-level characteristics and context and not focusing on “one-size-fits-all” interventions. Our findings align with the claims by Dugas et al [76] in that it is important to incorporate person-level characteristics in the design phase and set them up with the right type of mobile health intervention.

Our deeper-dive co-design sessions with Latinx families highlight the tensions that emerge between families and mobile health designers. Through co-design, we were able to understand how BIPOC families’ previous experiences shaped what they thought about sunscreen and sun protection, how culture plays a role in influencing their ideas about sun protection, and how children and their parents differ in design priorities. Co-design participating families wavered between dimensions from bilingual to monolingual and immigrants to first-generation US-born individuals.

Finally, the design features of our final intervention technology maximally reflected the themes that emerged during the co-design sessions. Our own designers had to make tough decisions about the mobile health innovation based on practicality and feasibility (eg, the use of budget Android phones), budget constraints, time to build and implement, what was technologically possible (eg, augmented reality and geofencing), real-world use by families, and privacy and security concerns. Although brilliant ideas came from the families, some of the ideas proposed via co-design were not possible to implement given the current technology or were beyond budget limits (eg, sensor-enabled automated talking curtains). We as health behavior intervention researchers also learned that users might interpret some of the survey questions that assess individual skin cancer risk as offensive and inappropriate (eg, skin tone description with a certain color name) and a violation of privacy and confidentiality (eg, “Why do you need to know what my eye color is?”). We resolved this issue by including additional information along with certain survey questions to assess skin cancer risk on the same page.

Finally, our use of the value tension framework [31,32] in this study is a contribution for public health researchers. Theoretical contributions in HCI can inform what we do, why we do it, and what we expect [77]. Theories and frameworks are used to distill a phenomenon into its essential features. We used the value tension framework to help sort our participants’ co-design responses into clear and easy-to-understand design guidelines. In our case, we had three populations engaging in complex co-design interactions: (1) parents and guardians, (2) children, and (3) design researchers. As these 3 groups were interacting so closely together, we believe it was important to discern the negotiated needs of the group.

Using the value tension framework, we were able to sort out the tensions between (1) children and adults, (2) family socioeconomic and health wellness needs, and (3) researchers and participants while being able to make specific design decisions from this organized view. Theoretical contributions in HCI are evaluated based on novelty, soundness, and predictive power [77]. In this case, the value tension framework can support public health researchers who may consider co-designing with populations that have complex ecological tensions (eg, people with disabilities, urban or rural populations, people with chronic illnesses, and senior citizens), particularly regarding health and wellness needs. This theory allows public health researchers to simplify and organize qualitative data into categories that can later become design guidelines.

### Limitations

The specific themes that we identified might not be generalizable to other community groups or to other health behaviors, and the challenges identified in our analyses might also be unique given the unprecedented temporal context of COVID-19. For instance, we did not anticipate going to the web for co-design in our initial plans in 2019. Despite these limitations, our current work highlights important and practical lessons for developing digital interventions for health behaviors.

JITAIs in mobile health can be designs that support behavior change within a changing and dynamic contextual state [1]. At the same time, the design of JITAIs in mobile health depends on multiple strategies within development. Specifically, continued work with historically marginalized families through co-design can be a way to support a deeper understanding of the tensions surrounding technology innovations for sun safety. Our study’s examination of co-designing with Latinx families through co-operative inquiry regarding SunSmart technologies revealed different kinds of value tensions that exist and need consideration. Through our analysis of families in 2 modes (local and web-based co-design), we were able to consider the value tensions that exist and respond through design decisions we made in our SunSmart app.

### Conclusions

In conclusion, by providing empirical data in co-design, we demonstrated the differing values among multiple BIPOC stakeholders, researchers, and designers. The use of the value tension framework allowed us to organize and make sense of the diverse voices in the design of JITAIs in mobile health [32]. Finally, our design implications surrounding the development of JITAI mobile interventions for BIPOC families can support designers and researchers who might consider the design dilemmas that take place in these kinds of community-based research opportunities. If mobile health and JITAI designers want to account for human values in the design of new innovations, we recommend explicit and intentional purposes to listen to and communicate with stakeholders on both conflict and opportunity in design.

Future work in this area needs to consider co-design as a strategy that can triangulate different design methods for JITAI mobile health in different groups beyond sun-protective behavior. We recommend that mobile health researchers consider co-design with value tension analysis with different stakeholders. It is important to consider our methods and analytical framing among marginalized groups that often do not have a chance to make direct inputs into advanced technologies. Furthermore, future work can also consider different ways to create new design methods and techniques to address tensions in design directly and in situ with marginalized groups. New ways to document emerging tensions and present them to stakeholders in the ongoing design process are important means to consider reflexivity in our methods.

## Abbreviations

BIPOC: Black, Indigenous, and people of color
HCI: human-computer interaction
JITAI: just-in-time adaptive intervention
LA: Los Angeles
PD: participatory design
RQ: research question
SES: socioeconomic status
SPF: sun protection factor

## Data Availability

The qualitative data sets (eg, co-design videos, artifacts, and analytic memos) generated and analyzed during this study are not publicly available because of the sensitive nature of the responses. The participants of this study were from a historically marginalized and susceptible population (Latinx English language learners and children), and we prioritized their confidentiality and privacy.
